# Disruptions to Care and the Use of Telehealth Among Older Adults During the COVID-19 Pandemic

**DOI:** 10.1093/geroni/igaa057.3452

**Published:** 2020-12-16

**Authors:** Beth Prusaczyk, Brian Carpenter, Nancy Morrow-Howell, Eric Lenze

**Affiliations:** 1 Washington University School of Medicine in St. Louis, Saint Louis, Missouri, United States; 2 Washington University in St. Louis, St. Louis, Missouri, United States

## Abstract

Reports emerged early in the 2020 COVID-19 pandemic that older adults were foregoing non-COVID19-related care, due to fears of contracting the virus during appointments and because of healthcare providers’ reduced operations. Beginning in July, 2020, we explored the impact of the pandemic on disruptions to care and older adults’ use of telehealth. Preliminary results from 53 older adults aged 66 to 93 (mean: 72.6) found that many older adults experienced disruptions in their care, ranging from 30-50% depending on the type of care. The most commonly disrupted care types were mental health and rehabilitation care (occupational, physical, or speech therapy), with 50% of older adults reporting disruptions to mental health and 50% to rehabilitation care. The most common reason for the disruptions was closed care providers’ offices. Similar results were found for primary care (46% reporting disruptions), dental care (44%), and vision care (30%), with between 62-71% citing closed offices as a reason for the disruption. The use of telehealth among the sample was high (44%), and the majority (83%) of these older adults reported never having used it previously. All who used it reported being very or somewhat comfortable with the technology, and 83% said they would use it again even if in-person care was available. These findings suggest the pandemic has had a significant impact on older adults’ care and that the expansion of telehealth could be increase access to care during and after the pandemic.

